# Machine Learning for Outcome Prediction of Acute Ischemic Stroke Post Intra-Arterial Therapy

**DOI:** 10.1371/journal.pone.0088225

**Published:** 2014-02-10

**Authors:** Hamed Asadi, Richard Dowling, Bernard Yan, Peter Mitchell

**Affiliations:** Melbourne Brain Centre, Department of Medicine, Royal Melbourne Hospital, University of Melbourne, Parkville, Victoria, Australia; Universitat Rovira i Virgili, Spain

## Abstract

**Introduction:**

Stroke is a major cause of death and disability. Accurately predicting stroke outcome from a set of predictive variables may identify high-risk patients and guide treatment approaches, leading to decreased morbidity. Logistic regression models allow for the identification and validation of predictive variables. However, advanced machine learning algorithms offer an alternative, in particular, for large-scale multi-institutional data, with the advantage of easily incorporating newly available data to improve prediction performance. Our aim was to design and compare different machine learning methods, capable of predicting the outcome of endovascular intervention in acute anterior circulation ischaemic stroke.

**Method:**

We conducted a retrospective study of a prospectively collected database of acute ischaemic stroke treated by endovascular intervention. Using SPSS®, MATLAB®, and Rapidminer®, classical statistics as well as artificial neural network and support vector algorithms were applied to design a supervised machine capable of classifying these predictors into potential good and poor outcomes. These algorithms were trained, validated and tested using randomly divided data.

**Results:**

We included 107 consecutive acute anterior circulation ischaemic stroke patients treated by endovascular technique. Sixty-six were male and the mean age of 65.3. All the available demographic, procedural and clinical factors were included into the models. The final confusion matrix of the neural network, demonstrated an overall congruency of ∼80% between the target and output classes, with favourable receiving operative characteristics. However, after optimisation, the support vector machine had a relatively better performance, with a root mean squared error of 2.064 (SD: ±0.408).

**Discussion:**

We showed promising accuracy of outcome prediction, using supervised machine learning algorithms, with potential for incorporation of larger multicenter datasets, likely further improving prediction. Finally, we propose that a robust machine learning system can potentially optimise the selection process for endovascular versus medical treatment in the management of acute stroke.

## Introduction

### Stroke and Endovascular Treatment

Stroke is a major global public health issue and is considered the third most costly health condition in developed countries [1]. Approximately 800,000 cases of stroke are reported in United States of America per annum, leading to 200,000 deaths, almost one of every 16 deaths [2], [3]. For those who survive, it is the most common cause of adult disability in the modern world [2], [4], requiring expensive long term rehabilitation care[2], [5]–[7] amounting to costs estimated at over 60 billion dollars per year in the United States of America alone [2], [5], [8]. More than 80% of stroke cases are ischaemic, with the remainder being haemorrhagic [2].

Urgent reperfusion of the ischemic brain is the primary treatment aim, either by intravenous thrombolysis or by endovascular interventional techniques [9]. These treatments focus on vascular recanalisation and restoration of blood flow to the ischemic tissue [10]. Although there are varying estimates to the potential number of patients who may benefit from endovascular intervention, there will likely be expansion of the number of patients treated using these techniques [2], [11], [12].

Initial focus was on intraarterial thrombolysis, proposed to be safe up to 6 hours post ictus in Pro-Urokinase for Acute Cerebral Thromboembolism II (PROACT-II) trial [1], [13]; however, rapid mechanical clot extraction with decreased time to cerebral reperfusion has obvious appeal and in fact is theoretically ideal for platelet poor, fibrin rich, well organized cardiogenic emboli, refractory to mechanical lysis^2^. Therefore, subsequent developments of various mechanical thrombectomy devices has gained much interest with the theoretical advantage of faster recanalisation and potential lower rate of hemorrhagic transformation; possibly leading to an extended time window in stroke intervention [1].

FDA approval for MERCI Retrieval and Penumbra Stroke Systems[2], [14]–[19] as the first generation of mechanical thrombectomy devices was followed by introduction of Solitaire and Trevo as stent retrievers [10]. Introduction of these devices backed up by the pioneering studies MERCI, multi-MERCI, and Penumbra, as well as Pivotal and SWIFT, have further strengthened the importance of the mechanical techniques in large vessels occlusion[10], [20]–[26]. However, despite more than 80% recanalisation success, randomised controlled trials such as Interventional Management of Stroke (IMS) - III [1], [27], have still failed to show a significant improvement in the clinical outcome, evaluated by 90 day modified Rankin Scale score[2], [10], [22], [28]–[33].

The SYNTHESIS trial also failed to show any superiority of endovascular intervention or even for combined endovascular and peripheral thrombolysis over traditional intravenous tPA[34]–[36]. This conundrum was made more complicated when the MR-RESCUE trial demonstrated not only that embolectomy was no better than standard care, but also a favorable penumbral pattern on imaging does not necessarily indicates patients who would benefit from endovascular therapy [37].

This discrepancy between the IMS-III, SYNTHESIS and MR-RESCUE outcomes and what may have been intuitively expected is likely related to the multiple potential pitfalls in the design of these trials which could change the interpretation of the results [1], [38], [39]. The most commonly speculated factor is that patient selection was neither targeted to those who failed IV thrombolysis, nor to those with large vessel occlusion or large clot burden ≥8 mm, who are usually not responsive to chemical treatment alone; as no vascular imaging was required prior to inclusion into the studies[1], [38]–[40]. These limitations could certainly influence the accuracy of the studies in evaluation of the clot retrieval techniques. On the other hand, stentretrievers, now acknowledged as more effective devices, were included only very late into the studies like IMS-III, with less than 1% of cases treated using Solitaire. Since the release of these preliminary results at least six additional devices have started premarket testing[1], [38]–[40].

It appears that the situation is different for posterior circulation involvement. Although causing only 6–10% of large vessel strokes, posterior circulation occlusions have a relatively different course, and failure of recanalisation, in particular in comatose patients or those with basilar trunk involvement results in a very poor prognosis [2], [41]. The BASICS (Basilar Artery International Cooperation Study) did not show a definite superiority for intraarterial intervention over intravenous thrombolysis [2], [42]; and the overall outcome is quite variable in patients who are treated with intraarterial or intravenous thrombolysis, in particular depending on the therapeutic delay [2], [43]. On the other hand, some trials have already demonstrate more than 50% recanalisation success rate for intraarterial techniques, with relatively good outcome [2], [44].

However, randomized control trials are restricted and limited by the lower incidence of posterior circulation strokes, and the results are potentially influenced by the heterogeneity of both the presentations and the causes; and at this stage the rational for aggressive treatment is mainly based on anecdotal evidence [2], [22], [23], [45].

Overall the major obstacle in endovascular intervention of the ischemic stroke is to establish a set of criteria identifying those patients who may benefit from intervention, whilst avoiding potential unwanted catastrophic treatment related complications.

There is currently level I evidence that NIHSS (National Institute of Health Stroke Score) [46], [47] is a quick and relatively simple guide to estimate the extent and the severity of a stroke, and probably correlates with the clinical outcome [46]. It is however, unable to measure the size of established infarction, separate from the salvageable parenchyma, and is therefore unable to predict potential outcome after endovascular intervention accurately. This is consistent with the well known fact that multiple factors contribute to and influence recanalisation success, including the extent and site of the vascular occlusion; and that the overall outcome also depends on patient demographic factors as well as clinical setting such as the time from onset, duration and the severity of the presenting neurological insult [10].

The complexity of the all of these factors involved, makes prediction of the final outcome difficult. On the other hand, undoubtedly, accurately predicting the outcome from a set of predictive variables is an important aspect of clinical work, which can assist in identifying high-risk patients and guide treatment approaches, thus potentially decreasing morbidity and mortality. Such a model in prediction of the outcome, not only may be crucial in prognostication, but can also have future roles in patient selection for the variety of the treatment options available and the relevant studies.

### Prognostic Modeling and Machine Learning

The usual approach to analyse the stroke outcomes data is to develop logistic regression models; however, machine learning algorithms have been proposed as an alternative, in particular for large-scale multi-institutional data, with the advantage of easily incorporating newly available data to improve prediction performance [48], [49].

Machine Learning algorithms can be applied and its trained, under two major different scenarios; supervised and unsupervised. In supervised scenario the predicted outputs are known and used to train the models. In unsupervised machines, the desired output is unknown, and the objective is to discover structure in the data, not to generalise a mapping from inputs to outputs [49], [50].

Two of the most commonly used machine learning methods include artificial neural network and support vector machine. These models are trained supervised, with neural network algorithms capable of unsupervised training as well [48], [49].

Although the technical details of theses algorithms are beyond the scope of this article, a summary of them follows:

Artificial neural network is a mathematical and computational model that is inspired by the structure and functional aspects of biological neural systems [49], [50]. It consists of interconnected nodes, processing information using a connectionist computational approach. The central connectionist principle proposes that complex neurological and mental phenomena can be described by an interconnected network of simple uniform units [50], adaptively changing their structure based on external or internal information, which flows during the learning phase, forming a robust dynamic system modelling the complex relationships between inputs and outputs or patterns in data[49]–[51].

From the different topological types of neural networks, the commonly used feed-forward is a network where connections between the units do not form a directed cycle or loop, and the information moves in only one direction, forward, from the input nodes, through the hidden nodes to the output nodes [50]. Back propagation algorithm is a supervised learning method divided into propagation and weight update phases, which are repeated until the performance of the network is good enough, while the output values are compared with the correct answer to compute the value of some predefined error-function [49], [50]. This calculated error is then fed back through the network, adjusting the weights of each connection accordingly, in order to reduce the error function [50]. Repeating this process usually eventually converges to some state where the error of the calculations is minimised, at which point the network is considered trained for a certain target function [49].

In comparison to the artificial neural network, the support vector machine works very differently. It takes a set of input data and predicts which of the different possible classes comprises the input, making it a non-probabilistic linear classifier. A set of training data is given and marked as belonging to one of the categories. An SVM training algorithm builds a model that assigns new data into one category or the other.

The example data points are initially mapped as points in space, so that the examples of the separate categories are divided by a clear gap that is as wide as possible, and then unknown data is represented in that same space, and predicted to belong to a category based on which side of the gap they fall on[49]–[51]. In doing so, the algorithm constructs a hyperplane or a set of hyperplane in an infinite-dimensional space, which can be used for classification, regression, or other tasks. Intuitively, a good separation is achieved by the hyperplane that has the largest distance to the nearest training data points of any class[49]–[51]. This gap is called functional margin, and in general the larger the margin the lower the generalisation error of the classifier[49]–[51].

Whereas the original problem may be stated in a finite dimensional space, it often happens that the sets to discriminate are not linearly separable in that space. For this reason, it was proposed that the original finite-dimensional space be mapped into a much higher-dimensional space, presumably making the separation easier in that space[49]–[51]. To keep the computational load reasonable, the mapping is designed to ensure that dot products may be computed easily in terms of the variables in the original space, by defining them in terms of a kernel function K(x,y) selected to suit the problem. The hyperplanes in the higher dimensional space are defined as the set of points whose inner product with a vector in that space is constant[49]–[51].

### Our Study

We aimed to design a prognostic model for the endovascular intervention in acute ischemic stroke using machine learning algorithms. We compared and assessed these two advanced methods in terms of their capability in predicting outcome.

We decided to separate anterior and posterior circulation strokes and model them independently to avoid potential inadvertent underlying inhomogeneities.

## Methods

*This is a retrospective study on a prospectively collected completely de-identified clinical database, which received approval from the ethics committee at our institution, and our review board has waived the need for consent (HREC: QA2011100). The technical details is provided below to facilitate reproducibility for other datasets if available.*

Demographics and clinical details of 107 patients who presented with acute anterior or posterior circulation stroke to our institution who underwent endovascular treatment, over a period of approximately five years, were extracted from a prospectively maintained stroke database (Table 1).

**Table 1:** Demographics and gender ratio.

Age and gender distribution of the patients:			
Age		Gender	
Mean	65.3	Female	41
Median	67	Male	66
Mode	80		
Std. Deviation	13.8		
Minimum	23		
Maximum	90		

Patients were all screened for relevant comorbidities at the time of presentation, including: diabetes mellitus, hypertension, hypercholesterolaemia, atrial fibrillation, history of ischemic heart disease and previous cerebral stroke or transient ischaemic attack. Neurological examination was performed for all of the patient prior to any intervention and the baseline National Institute of Health Stroke Scores were recorded in the database.

From the initial diagnostic angiogram occluded vessels were identified (Table 2). In the case of multiple sequential occlusions, the proximal vessel was used as a data point, and depending on the extent and segments involved, the artery was categorised as first, second and third occlusion.

**Table 2:** Distribution of the occluded vessels.

Occluded Arteries:			
Artery	Occlusion		
	1st	2nd	3rd
ACA	0	2	1
MCA- M1	55	21	0
MCA- M2	14	9	2
MCA- M3	1	1	0
MCA/ICA	1	0	0
ICA- Proximal	1	0	0
ICA- Intracranial/distal	22	2	0
ICA- extracranial	11	1	0
ICA- Terminal	1	0	0
CCA	2	0	0

Some of the patients also had IV-tPA prior to endovascular intervention. Different endovascular recanalisation devices were used, including Solitaire stent retriever, MERCI and Penumbra devices.

In addition to mechanical thrombectomy, some cases also received intraarterial chemical thrombolytic agents; and if present, associated or post-recanalisation hemodynamically significant stenoses were also treated with angioplasty or stent insertion.

After treatment of the occluded artery(s), recanalisation success was scaled using Thrombolysis in Cerebral Infarction (TICI) Score, by the blinded consensus of the treating neurointerventionalists. TICI score in conjunction with the number of attempts for recanalisation, procedure duration, and time of onset to recanalisation, as well as patient general anesthesia status, were all recorded into the database.

All procedural or delayed post-procedural complications were also recorded, including: arterial perforation and puncture site haematoma or pseudoaneurysm.

Post-procedure CT scans of the brain at 24–36 h were all assessed by neuroradiologist and neurointerventionists assessing for the presence of acute stroke and intracranial haemorrhage. Intracranial haemorrhagic transformations, were divided into clinically silent or symptomatic, and then classified into different categories (Table 3).

**Table 3:** ICH classification.

Classification of Infarction Haemorrhagic Transformation				
Type			Name	Definition
Asymptomatic		HI-1	Haemorrhagic infarction type 1	Small petechiae along the margins of the infarct
		HI-2	Haemorrhagic infarction type 2	More confluent petechiae within the infarcted area but withoutspace-occupying effect
		PH-1	Parenchymal haemorrhage type 1	Haematoma in ≤30% of the infarcted area with some slightspace-occupying effect
		PH-2	Parenchymal haemorrhage type 2	Dense haematoma in >30% of the infarcted area with substantialspace-occupying effect or as any haemorrhagic lesionoutside the infarcted area.
Symptomatic			Symptomatic intracranial haemorrhage	parenchymal haemorrhage type 2 (PH-2) withneurological deficit
Others	IVH		Interventricular Haemorrhage	
	SAH		Subarachnoid Haemorrhage	

Procedural outcome was monitored with modified Rankin Score, measured 90 days after onset. A final dichotomised good and bad outcome was also recorded for the patients as per mRS, with less than or equal 2 considered as good.

First, using SPSS® (IBM Corporation), a Standard Linear Model was designed, using Forward-Stepwise as the model selection method, and Information Criterion (AICC) as the criteria for entry. Potential predictors of the mRS as the outcome measure was identified and a prediction model was formed and compared with the observed outcome for validation.

Supervised machine learning was then attempted. Initially using MATLAB® (MathWorks Inc.) and its Neural Network Toolbox, a two-layer Feed-Forward network with sigmoid hidden and linear output neurons, was designed.

The data was then randomly divided into 70, 15 and 15 percents subsets and the network was trained using Levenberg-Marquardt algorithm, validated and tested using the modified Rankin score as outcome; with the performance of the model monitored using Mean Squared Error. Prediction errors were also depicted on a histogram.

In addition, for comparison, the network was also trained using the dichotomised mRS, >2 or ≤2, to evaluate a binary classifier for potential good and poor outcomes.

For the seven scale mRS network, linear regressions were also performed between the observed and estimated outcome, over the training, validation and test datasets independently using Theil–Sen estimator.

However, with the dichotomised model being a binary classifier, Receiver Operating Characteristic curves were calculated to illustrate the performance of the system over each dataset as its discrimination threshold is varied. In addition, confusion matrices or contingency tables were also calculated, allowing better representation of the performance of the network.

The designed network and its calculated weighting matrix was then saved to be imported into the Simulink Toolbox of MATLAB® (MathWorks Inc.) for outcome prediction of the future data.

Subsequently to assess the capabilities of other supervised machine learning systems, the dataset with scaled and dichotomised mRS were imported into the data-mining program, Rapidminer® (Rapid-I Inc.). The filtered data was then given to the input training port of a nested cross-validation operand, with the relative number of validation of 10% and a shuffled sampling type, as well as “Leave One Out”.

The cross-validation operand consisted of two components, training and testing. The testing component contained a Support Vector Machine, with ANOVA Kernel, which is defined by raised to power “d” of summation of “exp(−g (x–y))” where “g” is gamma and “d” is the degree. “g” and “d” were set to be 1 and 2 in our machine.

The size of the cache for kernel evaluations was set to be 200 megabytes. The complexity constant (“C”) which sets the tolerance for misclassification, was set to be zero. The convergence epsilon, which is an optimizer parameter specifying the iterations stop point, was set to 0.001, with maximum iteration set to be 100000. In our machine, the loss function positive and negative complexity constant was set to 1.0. The insensitivity constant, epsilon as well as the epsilon for positive and negative deviations, were all set to be zero.

The model calculated in this machine is passed onto the testing component of the parent x-validation operand and then applied on to the test dataset.

The performance of the machine was monitored by a classic performance monitor operand and was reported as the mean squared error as well as its root. In addition, accuracy of the machine was also assessed by aggregation of a hidden confusion matrix constructed by evaluating different models on different test sets.

The designed model is finally incorporated into an apply operand ready for the prediction of the outcome of the future patients.

## Results

Average of the patients’ baseline stroke score was 17.7 (SD: ±7.9).

44 of our patients also had IV-tPA prior to endovascular intervention. The remainder of the patients did not receive tPA due to a variety of contraindications.

81% of the procedures were performed under generalized anesthesia, and our three neurointerventionalists (BY, PJM and RJD) performed 24, 48, 35 cases respectively, as the primary operator.

Solitaire stent retriever was used in the majority of the cases 73, MERCI and Penumbra devices were used in 17 and 2 cases respectively. In some cases instrumentation was repeated up to 6 times to improve recanalisation.

2 cases received urokinase, 4 tissue plasmin activator, and 4 plasmin, as adjunct intraarterial chemical thrombolytic agents.

Overall recanalisation has been very successful with TICI 2b or 3 demonstrated on the final angiographic run in approximately 50% of cases. Significant associated and post-recanalisation arterial stenosis was also noted in some cases, with 23 patients requiring angioplasty, with 10 patients eventually stented.

On average procedures have taken 82.3 minutes (SD: ±39.0). The time of onset to recanalisation was on average 339.7 minutes (SD: ±91.8).

Immediate procedural complications including arterial perforation or puncture site haematoma and pseudoaneurysm were uncommon, only seen in 1 and 2 cases respectively.

31 patients were diagnosed with intracranial haemorrhage on the delayed post procedural CT, with a wide spectrum of locations and severities, from subarachnoid hemorrhage to asymptomatic or large intra-parenchymal bleedings (Table 4).

**Table 4:** Haemorrhagic Transformation.

Type		No.
Asymptomatic	HI-1	4
	HI-2	16
	PH-1	1
	PH-2	5
Symptomatic		5
Others	IVH	0
	SAH	0

**Figure 1:**
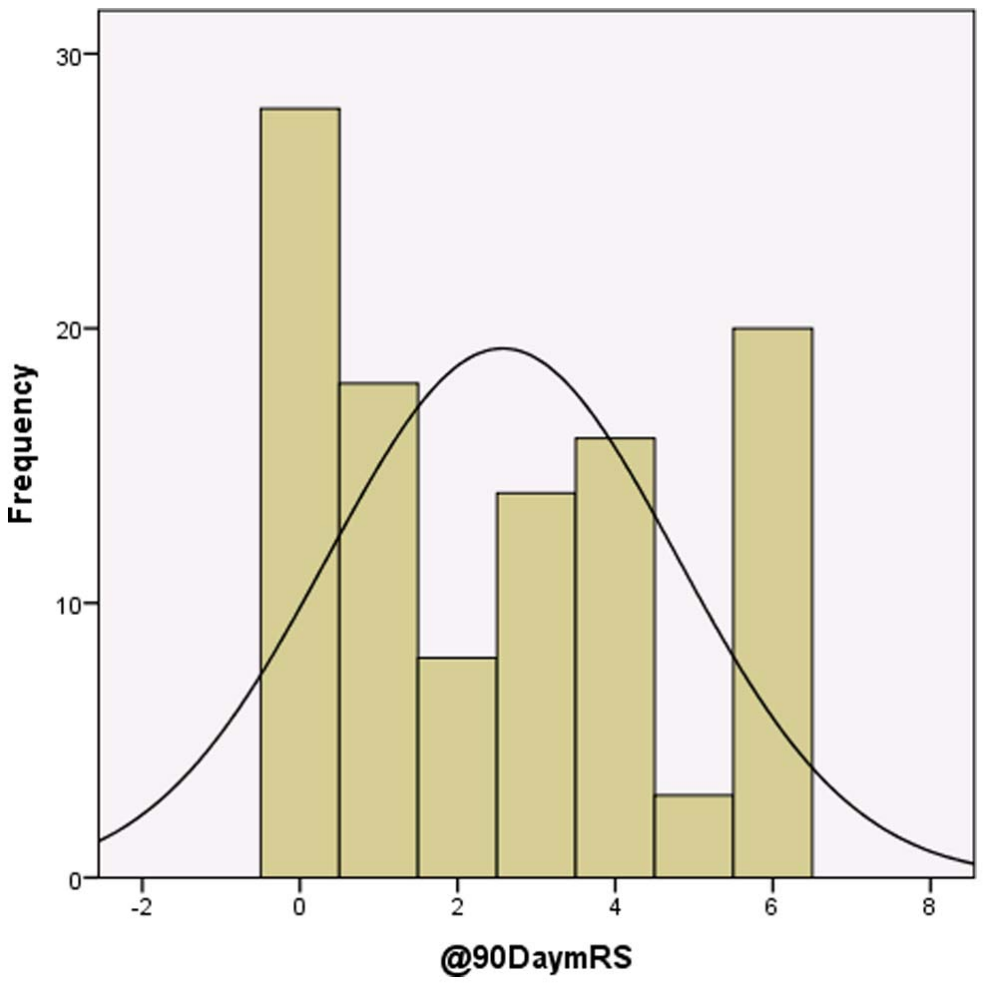
90 days mRS histogram.

The average of mRS at 90 days was 2.57 (SD: ±2.21), with median of 2 and mode of 0 (Figure 1 & Table 5).

**Table 5:** 90 days mRS.

Modified Rankin Score (mRS)	
Mean	2.57
Median	2
Mode	0
Std. Deviation	2.21
Minimum	0
Maximum	6

### Standard Modelling

**Figure 2:**
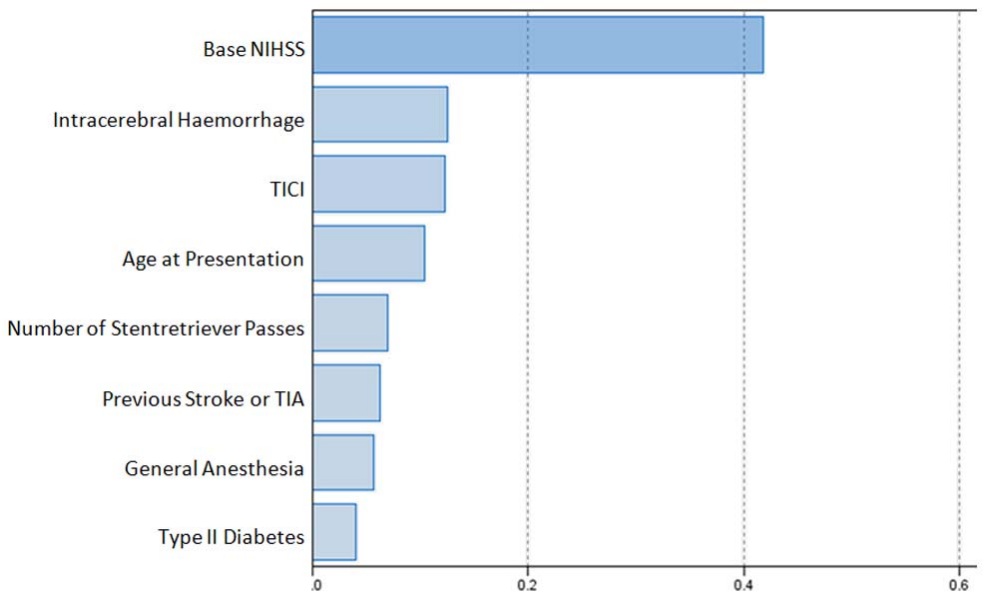
Relative importance of the model’s variables in prediction of mRS at 90 days (outcome).

**Figure 3:**
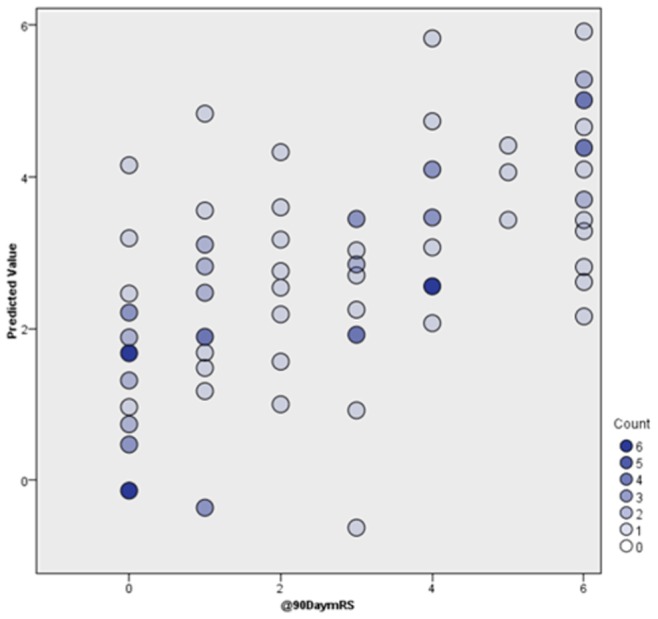
Comparison between predicted and observed outcome.

The information criterion and accuracy of the proposed linear model were calculated as 119.67 and 43.5% respectively (Figure 2). The most influential predictor was baseline NIHSS, with relative predictive value of 0.4 (Figure 3).

### Artificial Neural Network

**Figure 4:**
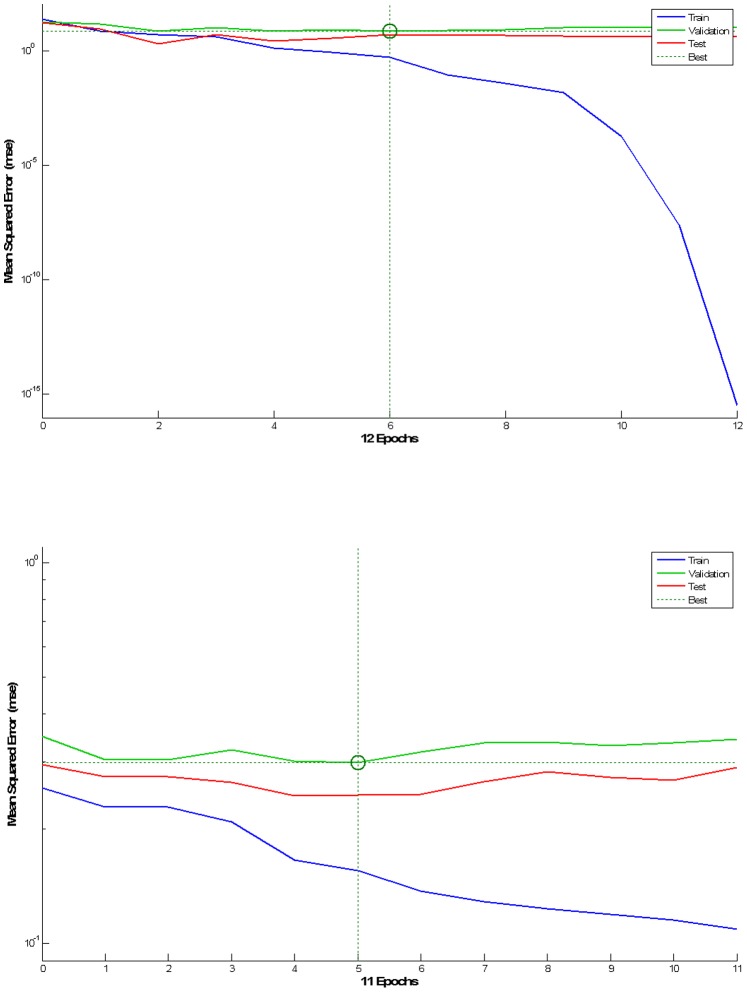
Network performance, for seven scale (left/top) and dichotomised mRS models (right/bottom). Legend: Blue-Training, Green-Validation, Red-Test, Dashed Lines-Best, Vertical Axis-MSE, Horizontal Axis-Epochs.

The best validation performance was 6.94 and 2.98, at epoch 6 and 5 for seven scale and dichotomised mRS models respectively (Figure 4).

Gradient of 1.305×10^−7^ and 3.038×10^−1^ were calculated at epoch 12 and 11 for seven scale and dichotomised mRS models respectively.

**Figure 5:**
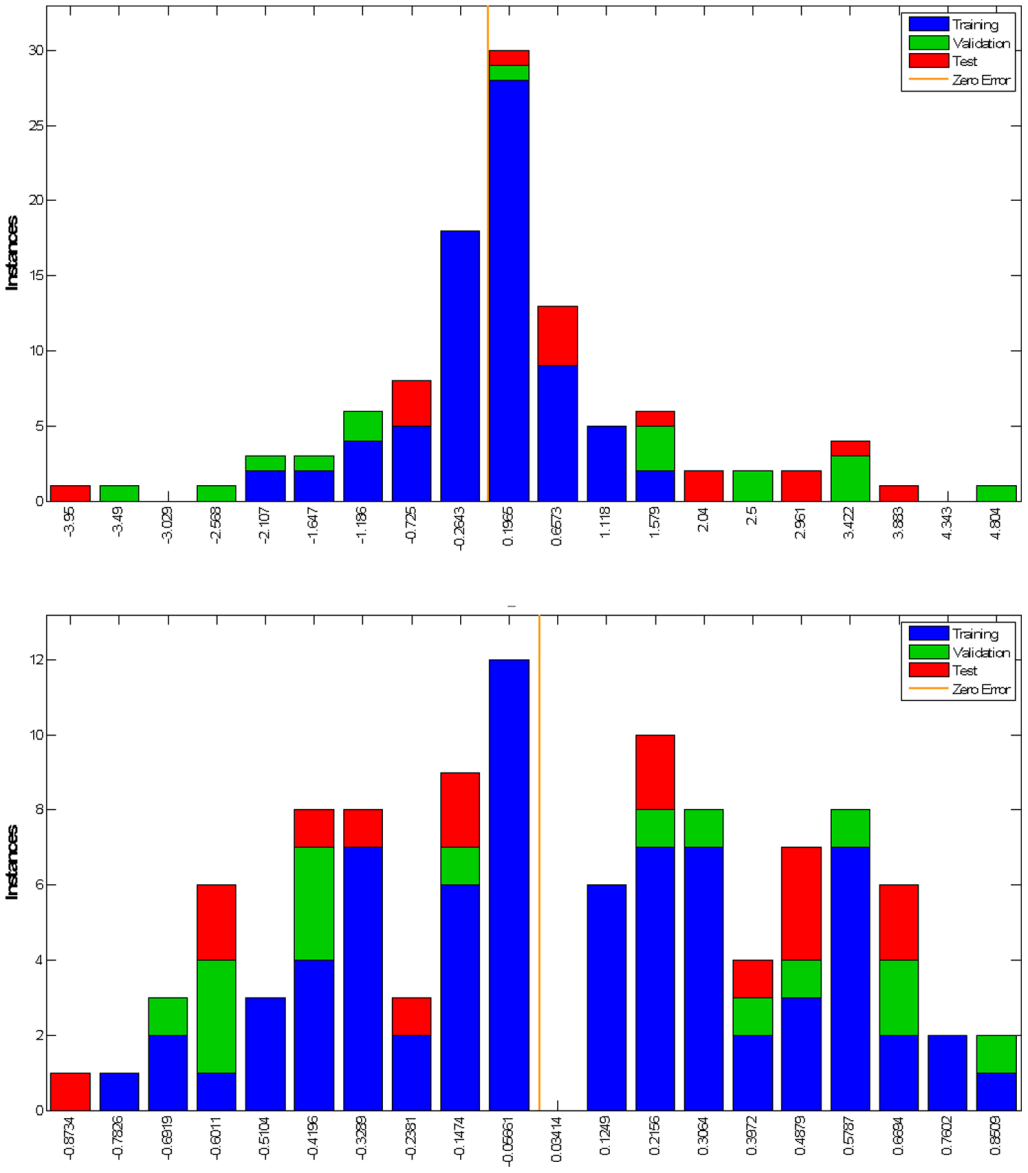
Error Histogram, for seven scale (left/top) and dichotomised mRS models (right/bottom). Legend: Blue-Training, Green-Validation, Red-Test, Orange Line-Zero Error, Vertical Axis-Instances, Horizontal Axis-Error.

Error histograms were calculated as the difference between the target and output which are equivalent of observed and estimated outcome, from the training, test and validation datasets, for seven scale and dichotomised mRS models (Figure 5).

**Figure 6:**
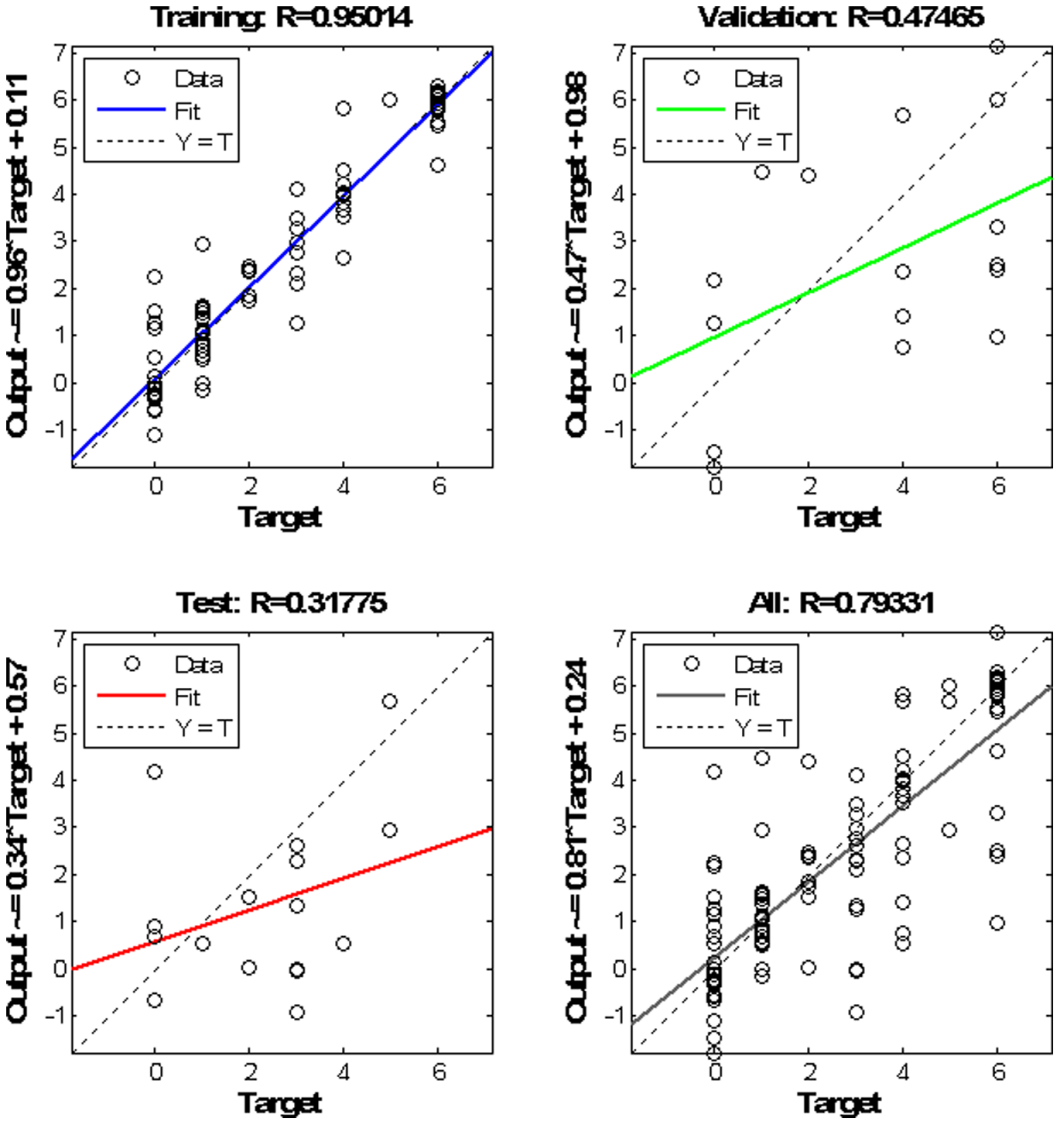
Linear fit between the estimated and observed outcome.

Using Theil–Sen estimator, the root of the Coefficient of Determination calculated as 0.95, 0.47 and 0.32 for each subset respectively. However, overall network estimated and observed outcome for the whole dataset, demonstrate a relatively good linear correlation with an R of 0.79 in a linear regression (Figure 6).

Overall, favourable ROC curves; however, the test ROC curve is relatively poor, with the estimated area under curve (AUC) of 0.6.

**Figure 7:**
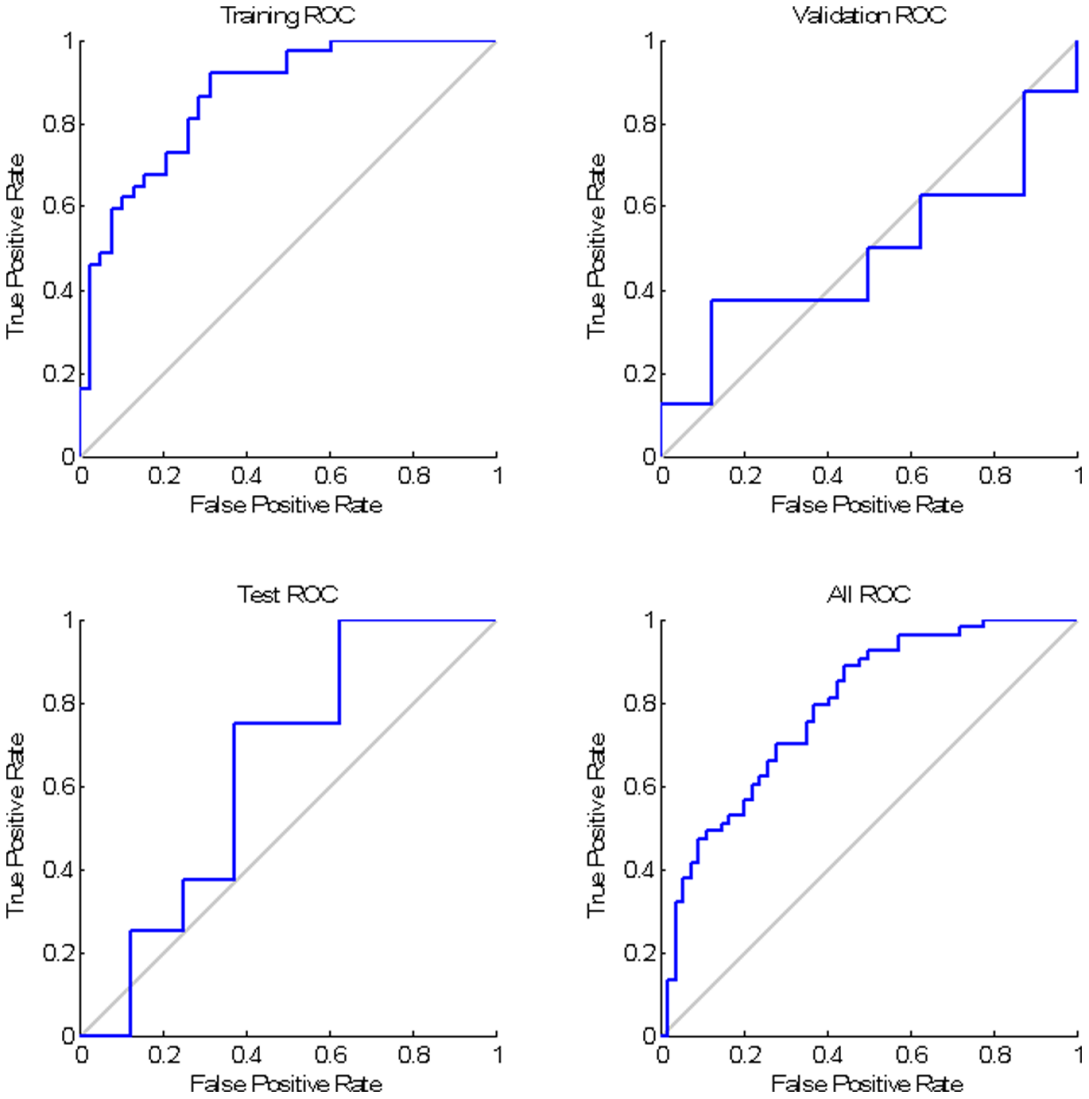
Over all ROC curve and Confusion Matrix for the dichotomised outcome network.

The contingency table, with each column representing the instances of the predicted outcome and each row demonstrating the observed outcome, confirming acceptable model sensitivity and specify (Figure 7).

### Support Vector Machine

For the scaled mRS outcome, our support vector machine, had a good performance with a mean squared error of 4.489 (SD: ±2.438) and the estimated root at 2.064 (SD: ±0.480). Also, the system accuracy was assessed by “mikro”, calculated as 2.128.

On the other hand, system’s MSE and “mikro” were calculated as 0.262 (SD: ±0.068), and 0.514, respectively in prediction of the dichotomised outcome, with the precision of 80% in prediction of the poor outcome and an overall precision of 87% and model accuracy of approximately 70% (Table 6).

**Table 6:** Model precision of support vector machine in prediction of the dichotomised intervention outcome.

Outcome	True Poor	True Good	Class Precision
**Predicted Poor**	27	7	79.41%
**Predicted Good**	26	47	64.38%
**Class Recall**	50.94%	87.04%	87.04%

Best performance of the scaled mRS model was in prediction of the patients’ prognosis with mRS of 3, with a class precision of 100% and the least precise prediction was for those patient with mRS of 6, which was only approximately 25%.

Estimated machine’s performance was improved even further when the cross validation operand set to work with “Leave Out One” sampling rather than “Shuffled”, with a MSE of 4.347 (SD: ±4.425) and rMSE of 1.809 (SD: ±1.037) for the scaled mRS outcome, and 0.286 (SD: ±0.367) and rMSE of 0.441 (SD: ±0.302) for the for the dichotomised model.

The “mikro” indicator of accuracy, was calculated as 2.085 and 0.535, with the “Leave Out One” sampling for the scaled mRS and dichotomised outcome predictor machines, respectively.

## Conclusions

We showed, despite a small dataset, that there was promising accuracy, approaching 70%, of predicting outcome. There is the likely potential of further improving prediction by the incorporation of larger multicenter datasets.

There has been recent interest in adopting machine learning techniques in the prediction of the outcome of stroke patients. A recent study proposed spatial regularisation of the diffusion-weighted images acquired at the acute stage using support vector machine with a Graph encoding the voxels’ proximity, and concluded significant accuracy in prediction of the motor outcome at 90 days, showing that poor motor outcome is associated with the changes in the corticospinal bundle and white matter tracts originating from the premotor cortex [52].

Another study has proposed use of machine learning in individualised stroke treatment decision making by accurate identification of the extent of salvageable tissue on MRI in rats, based on measurement of a perfusion-diffusion mismatch and calculation of infarction probability. This study has compared generalized linear model (GLM), generalized additive model, support vector machine, adaptive boosting, and random forest; proposing that assessment of the heterogeneity of infarction probability with imaging based algorithms enables estimation of the extent of potentially salvageable tissue after acute ischemic stroke [53].

Conversely, attempts to prove the effectiveness of the invasive stroke treatments have shown inconsistent results. However, more than ever before, endovascular treatments of acute ischemic stroke are opening their way into the mainstream management of the acute stroke, in particular for those patient with contraindication for IV thrombolysis or large vessel occlusions[10], [54]–[58].

To our knowledge there is no comprehensive multifactorial study in humans, attempting to apply machine learning algorithms in acute ischemic stroke outcome prediction, after invasive endovascular management. Undoubtedly numerous factors, including extensive clinical features, can influence the final stroke outcome with varying significance and mechanisms, making conventional modelling challenging and perhaps inaccurate.

Machine learning models however, being, relatively independent of the unknown potential underlying interactions between these factors, are probably able to simulate the eventual result of such a complex system.

Such models can be of extreme use not only for prognostication and in predicting the outcome under different circumstances, but also in near future as an assistant in clinical decision making in particular identifying those patients who may benefit from a variety of possible treatment options, including more aggressive management, like endovascular interventions.

### Limitations

Parallel to the all abovementioned advantages of the machine learning algorithms, there are important underlying assumptions and limitations that should not be forgotten. These models although can be accurate, and perhaps useful in answering the primary question, but more or less behave as a “black box” requiring large training datasets to improve their performance, with the true underlying relationships between influential factors remaining undiscovered to the user [48], [49], [51].

This inherent need for large training datasets may affect the accuracy of the machines in studies, like the current study, when only representative training data is used. In addition, with no clear understanding of the true predictors, an overcorrected conservative design may lead to the models being over-fitted by irrelevant demographics or clinical factors, thus increasing the random error and covering the desired signal with noise, a phenomenon which may explain the poor ROC curve for the test group, in this study. To avoid this, techniques like cross-validation, regularization, pruning or Bayesian model comparison, can be used to indicate the tipping point when further training no longer results in a better performance; or alternatively decision tree learning methods can be employed, providing more interpretable models [48], [49], [51].

### Future Work

**Figure 8:**
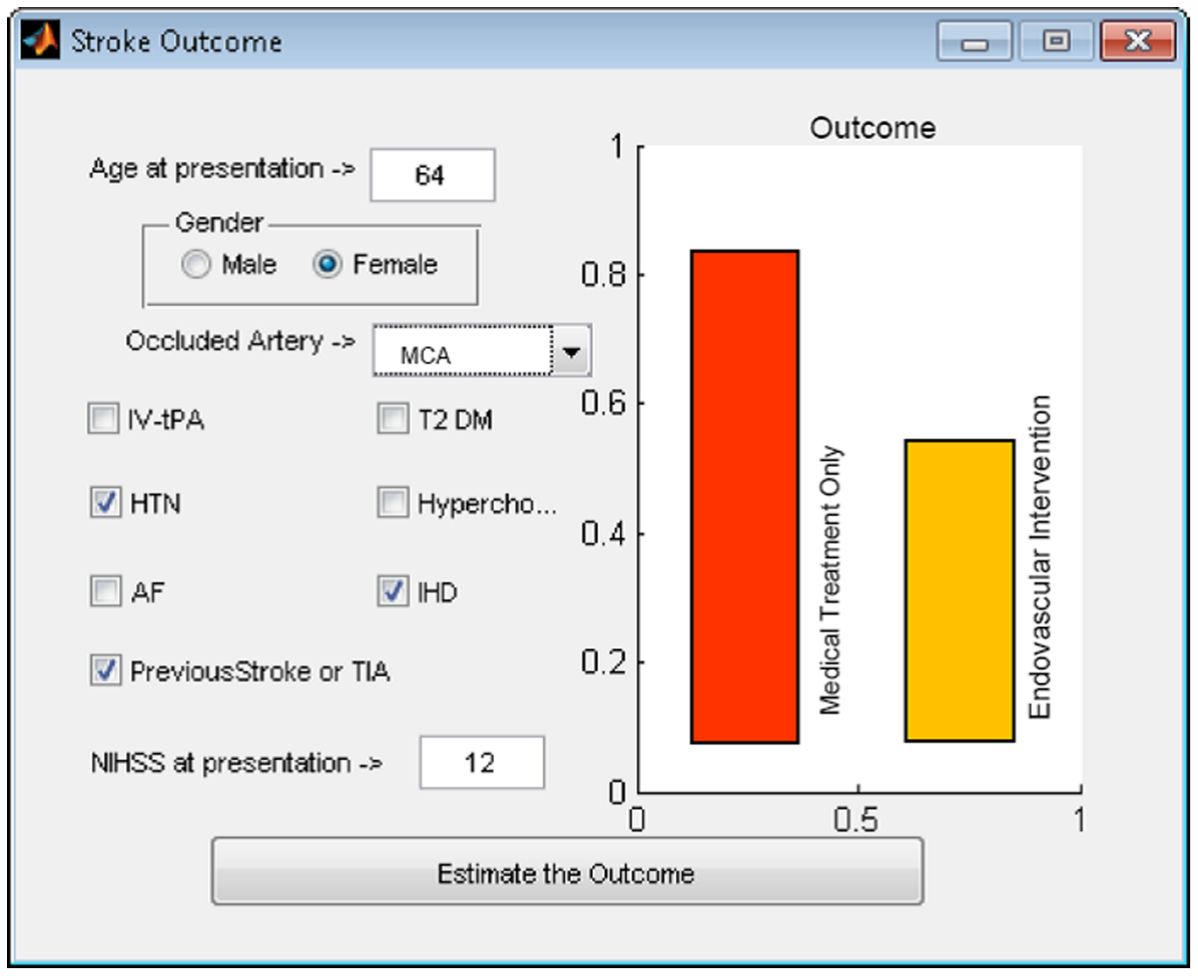
Ultimate goal is to design a system capable of proposing a dichotomised outcome for each patient with and without endovascular intervention.

All of the underlying methodological and computational complexities aside, our long term goal is to design an easy to use online system, allowing for relative prediction of the clinical outcome based on the demographics and clinical findings, which can be used as a guide in making appropriate therapeutic decisions (Figure 8).

Such a system has the potential for fine adjustment from the continuous training provided via handling large-scale national or international multi-institutional users, with the advantage of easily incorporating newly available data to improve prediction performance.
